# AI-Enhanced Analysis Reveals Impact of Maternal Diabetes on Subcutaneous Fat Mass in Fetuses without Growth Alterations

**DOI:** 10.3390/jcm12206485

**Published:** 2023-10-12

**Authors:** Hector Borboa-Olivares, Johnatan Torres-Torres, Arturo Flores-Pliego, Aurora Espejel-Nuñez, Ignacio Camacho-Arroyo, Mario Guzman-Huerta, Otilia Perichart-Perera, Omar Piña-Ramirez, Guadalupe Estrada-Gutierrez

**Affiliations:** 1 Community Interventions Research Branch, Instituto Nacional de Perinatología, Mexico City 11000, Mexico; 2 Clinical Research Division, Instituto Nacional de Perinatología, Mexico City 11000, Mexico; torresmmf@gmail.com; 3 Department of Immunobiochemistry, Instituto Nacional de Perinatología, Mexico City 11000, Mexico; arturo.flores@inper.gob.mx (A.F.-P.); aurora.espejel@inper.gob.mx (A.E.-N.); 4 Unidad de Investigación en Reproducción Humana, Instituto Nacional de Perinatologia-Facultad de Química, Universidad Nacional Autónoma de México, Mexico City 11000, Mexico; camachoarroyoi@quimica.unam.com; 5 Department of Translational Medicine, Instituto Nacional de Perinatología, Mexico City 11000, Mexico; mguzmanhuerta@yahoo.com.mx; 6 Nutrition and Bioprogramming Department, Instituto Nacional de Perinatología, Mexico City 11000, Mexico; oti_perichart@yahoo.com; 7 Bioinformatics and Statistical Analysis Department, Instituto Nacional de Perinatología, Mexico City 11000, Mexico; omar.pina@inper.gob.mx; 8 Research Division, Instituto Nacional de Perinatología, Mexico City 11000, Mexico

**Keywords:** diabetes and pregnancy, ultrasound evaluation, fetal subcutaneous fat mass

## Abstract

Pregnant women with diabetes often present impaired fetal growth, which is less common if maternal diabetes is well-controlled. However, developing strategies to estimate fetal body composition beyond fetal growth that could better predict metabolic complications later in life is essential. This study aimed to evaluate subcutaneous fat tissue (femur and humerus) in fetuses with normal growth among pregnant women with well-controlled diabetes using a reproducible 3D-ultrasound tool and offline TUI (Tomographic Ultrasound Imaging) analysis. Additionally, three artificial intelligence classifier models were trained and validated to assess the clinical utility of the fetal subcutaneous fat measurement. A significantly larger subcutaneous fat area was found in three-femur and two-humerus selected segments of fetuses from women with diabetes compared to the healthy pregnant control group. The full classifier model that includes subcutaneous fat measure, gestational age, fetal weight, fetal abdominal circumference, maternal body mass index, and fetal weight percentile as variables, showed the best performance, with a detection rate of 70%, considering a false positive rate of 10%, and a positive predictive value of 82%. These findings provide valuable insights into the impact of maternal diabetes on fetal subcutaneous fat tissue as a variable independent of fetal growth.

## 1. Introduction

Abnormal fetal growth is linked to higher rates of perinatal morbidity and mortality and an increased risk of metabolic diseases later in life, including diabetes, hypertension, obesity, metabolic syndrome, and dyslipidemia [1,2]. In pregnant women, pre-existing diabetes and inadequate metabolic control can negatively impact embryogenesis during early gestation and significantly influence growth and body composition later in pregnancy [3]. Poor glucose control in pregnancies complicated by diabetes, whether insulin-dependent or gestational, often results in identifiable characteristics such as selective macrosomia (excessive fetal growth) and organomegaly (enlargement of organs) [4]. Furthermore, diabetic pregnant women with complications such as preeclampsia or pre-existing vascular disease may experience reduced uterine flow and morphological changes in the placenta, which affect nutrient exchange, leading to intrauterine growth restriction [5].

Maternal hyperglycemia induces fetal hyperglycemia, stimulating pancreatic activity resulting in hypertrophy, hyperplasia, and increased insulin secretion. Insulin is the primary anabolic hormone for fetal growth and development, contributing to macrosomia and organomegaly [1,3,6,7]. Current evidence suggests that maintaining reasonable glycemic control in pregnant women with diabetes can disrupt the cycle of hyperglycemia and hyperinsulinemia, thus preventing complications associated with abnormal fetal growth [8,9]. However, it remains uncertain whether poor metabolic control in the latter half of pregnancy exclusively impairs fetal growth [10].

Changes in fetal body composition have implications for both the life period within the uterus and after birth, leading to alterations in metabolism and inflammation, increasing the fetus’s vulnerability to higher morbidity and long-term consequences [11,12]. As a result, evaluating fetal body composition provides numerous advantages over conventional methods used to assess fetal growth. Previous studies have investigated fat levels in fetuses of diabetic mothers, revealing elevated subcutaneous or abdominal fat areas [13,14,15]. However, these techniques for evaluating fat are impractical for routine clinical use due to their limited reproducibility attributed to operator bias involved in manually selecting the ultrasound plane for measurement [16,17,18,19]. Consequently, ongoing research aims to develop innovative tools capable of detecting changes in fetal body composition, enabling early and comprehensive assessments of growth disorders, and ultimately enhancing clinical management and perinatal outcomes.

Artificial intelligence (AI) has shown some benefits in clinical research. These tools in obstetrics have been used to incorporate data and images in machine learning models to predict preterm birth, birth weight, preeclampsia, mortality, hypertensive disorders, and postpartum depression and placental abnormalities, offering a reduction in inter- and intraoperator variability, time reduction in procedures, and improving overall diagnostic performance [20,21,22].

The present study evaluated subcutaneous fat tissue in fetuses with normal growth among pregnant women with well-controlled maternal diabetes using a more reproducible 3D-ultrasound tool and offline TUI (Tomographic Ultrasound Imaging) analysis [23]. Additionally, three artificial intelligence (AI) classifier models were trained and validated to assess the impact of maternal diabetes on subcutaneous fat mass in fetuses, identifying the offspring of a diabetic mother.

## 2. Materials and Methods

### 2.1. Ethics Statement

This study was conducted as part of the ongoing OBESO (Biochemical and Epigenetic Origins of Overweight and Obesity) perinatal cohort at the Instituto Nacional de Perinatologia (INPer) in Mexico City, which aims to investigate the association between obesity, maternal metabolic profile, and their predictive roles in fetal body composition, obesity, and neurodevelopment during infancy. The project was approved by the Ethics and Research Internal Review Board (2016-1-568/2017-2-79). Enrolled women were provided with detailed information regarding the risks and benefits of the study, and their participation was voluntary. Informed consent was obtained from all recruited participants.

### 2.2. Study Population

Sixty singleton pregnant women were conveniently selected during their third-trimester ultrasound appointments from January to December 2019. Thirty of these women had well-controlled diabetes, including sixteen with pregestational (type 2) diabetes without pre-existing vascular disease and fourteen with gestational diabetes. The other thirty women were selected as healthy controls, matched by gestational age. The control group underwent an oral glucose tolerance test between 24 and– 28 weeks of gestation to rule out diabetes. Patients used as controls were paired for gestational age, fetal gender, BMI classification (underweight, normal weight, overweight), and weight gain at the time of the study (adequate, insufficient, or excessive) [24]. The diabetic participants maintained good glycemic control throughout pregnancy based on the guidelines set by the American Diabetes Association, which included fasting capillary glycemia ≤ 95 mg/dL and one-hour postprandial capillary glycemia ≤ 140 mg/dL in at least 80% of measurements, with glycosylated hemoglobin HbA1c levels below 6.0% [25]. Women were enrolled after 31 weeks of gestation, as determined by the last menstrual period and confirmed by the first-trimester ultrasound. Participants with chronic or pregnancy-induced high blood pressure, type 1 diabetes, diabetes with vasculopathy, and intrauterine fetal growth alterations were excluded from the study. All enrolled women received routine prenatal care at INPer, and relevant clinical data were extracted from their medical records. Women with diabetes received medical nutrition therapy provided by a dietitian, and in some cases, pharmacological treatment with metformin was necessary to achieve adequate metabolic control. The Department of Endocrinology at INPer adjusted the metformin dosage to maintain optimal glycemic standards. Maternal anthropometric measurements, including pre-gestational weight, height, and body mass index, were obtained from the medical records. All patients included were followed up to pregnancy ended to collect perinatal outcomes. No sample size calculation was performed beforehand, but the statistical power was calculated for all variables with significant differences to verify that it was greater than 80%.

### 2.3. Fetometry

Fetometry was performed using a Voluson E8 (GE Healthcare, Chicago, IL, USA) 3D ultrasound with a volumetric transducer (4–8 MHz). Measurements such as biparietal diameter, head circumference, abdominal circumference, and femoral length were taken to estimate fetal weight using the Hadlock 2 formula. Weight percentiles were calculated based on gestational age, according to the Hadlock reference values, preloaded in the ultrasound machine; all fetuses included were weighed between the 10th and 90th percentile. The ultrasound examination involved acquiring a 3D volume scan with a 30° sweep angle and an acquisition time of 10 s. To ensure accuracy, the transducer was placed as close as possible to the extremity without applying pressure and with minimal fetal and maternal movement. The arm and thigh closest to the mother’s abdominal wall were selected for measurement.

### 2.4. Assessment of Fat Mass Area

Volumetry was performed on the arm and thigh (humerus/femur) anterior to the maternal abdominal wall, placing the transducer as close as possible to the limb without exerting pressure in the absence of fetal and maternal movement. The volumetry transducer was selected (4–8 MHz), and the initial settings were the same as used in the 2D evaluation; only contrast and zoom were increased in order to see the complete structure in 70 to 80% of the screen, the focus was placed in the area of interest and the gains to optimize the image. A volume acquisition angle of 80° was selected, and the limb was centered correctly. The quality of the images depended on the exposure speed; a rotational scan was selected in a sagittal Z plane with an acquisition time of 10 s [26].

In the offline evaluation, the ViewPoint program, GE Healthcare, was used; select the “explore submenu”, and then the acquired file was chosen. The sagittal plane of the bone was displayed as the main screen, and the proximal epiphysis lateralized to the left. A Sepia filter was applied in the image to delineate the lean and fat mass contours. In the TUI (Tomographic Ultrasound Imaging) tool, three tomographic slices were programmed, and the diaphysis was centered to have the center, 1 to the right and 1 to the left [26].

**Figure 1:**
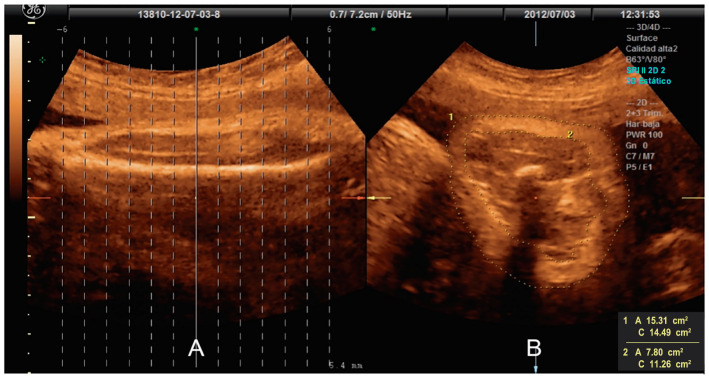
Offline Tomographic Ultrasound Imaging analysis. (**A**) Sagittal plane of the fetal femur. (**B**) Axial plane selected, fat area obtained by subtracting the lean tissue area (muscle and bone) from the total area (covering the total area).

The fat mass area was determined by subtracting the central area representing lean mass, consisting of bone and muscle, from the total area obtained in the image. At least two measurements were taken for each tomographic plane, and the average of these observations was used for analysis. Three planes of the humerus/femur were utilized: the union of the proximal third with the middle, the middle of the bone, and the union of the distal third with the middle third (Figure 1). The acquisition of images and the subsequent offline analysis were performed by three ultrasound experts specialized in maternal–fetal medicine, who followed a standardized technique. Prior to the study, the technique was standardized among these three operators. The inter- and intra-observer variability was calculated using the intraclass correlation coefficient, yielding a value greater than 0.90 for all three selected planes.

### 2.5. Statistical Analysis

Statistical analysis was performed using IBM^®^ SPSS^®^ Statistics, version 20, and descriptive statistics were employed to characterize the general population. The paired *t*-test and Wilcoxon rank test were used to assess differences based on data normality and the requirement for non-parametric tests, respectively. Statistical significance was considered for *p* < 0.05.

### 2.6. Classifier Models

Data analysis comprised two stages: (A) Feature Selection, during which relevant variables were identified using a 70-30% bootstrapping technique, and (B) Classifiers Training and Validation, which involved training and evaluating three Linear Discriminant Analysis with Shrinkage models via a cross-validation process. Each model accounted for a different group of features. For the classification task, control and diabetes case data were labeled with 0 and 1, respectively.

(A) Feature Selection

The Least Absolute Shrinkage Selector Operator (LASSO) is a regularized version of linear regression that assigns zero weight to non-relevant features and, therefore, serves as a feature selector.

**Figure 2:**
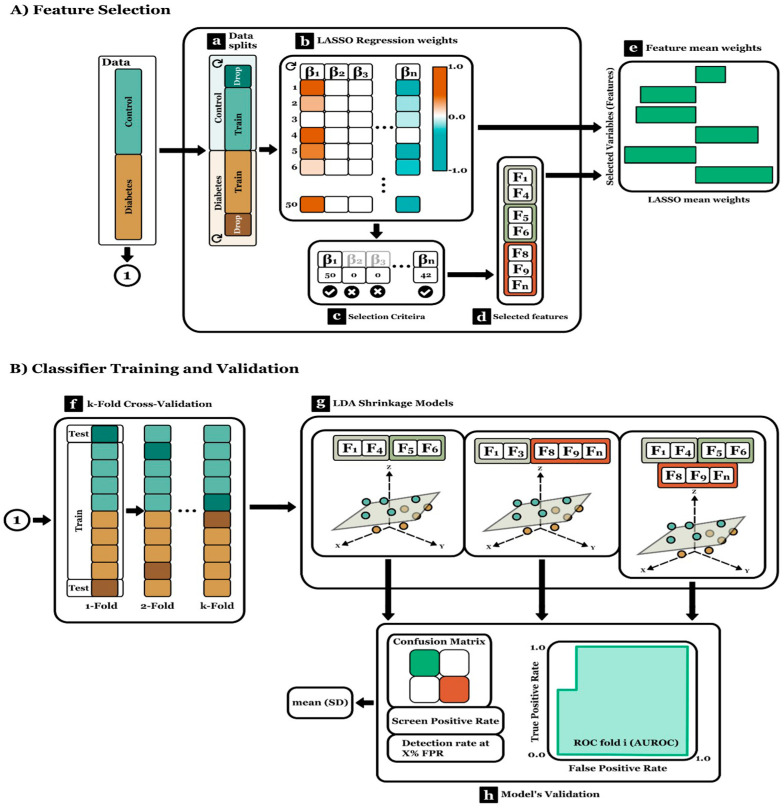
Classifier model development process, variable selection, training, and validation.

An optimal LASSO model was trained using 70% of the class-stratified data in each bootstrapping iteration. The remaining 30% was discarded as this stage focused on determining the variables that contributed the most relevant information (Figure 2a). Optimal LASSO models were obtained using Python’s sci-kit-learn library. This process was repeated across ten blocks, each comprising five bootstrapping iterations (Figure 2b).

Features were selected based on variables that did not register any non-zero-weight counts of LASSO (Figure 2c). These were grouped according to variable nature: biometric, free fat-mass, and fat-mass. The average weight of the features is displayed in bar plots (Figure 2e).

(B) Classifier Training and Validation

The classifier utilized was the Linear Discriminant Analysis, which employed a shrinkage approach with Ledoit–Wolf parameter optimization. Three models were trained, and each included a different combination of feature groups: biometric + free fat mass, biometric + fat mass, and biometric + free fat mass + fat mass (Figure 2g). Classification performances were assessed using a class-stratified 10-fold cross-validation technique (Figure 2f). Finally, the mean and standard deviation of the Area under the ROC (AUROC), Detection Rate adjusted with False Positive Rate percentage, and Screen Positive Rate are reported (Figure 2h).

Three artificial intelligence classifier models were trained and validated to assess the clinical utility of fetal subcutaneous fat measurement. Model 1, referred to as “full”, included the following variables: subcutaneous fat measured by ultrasound, gestational age, fetal weight (ultrasound), fetal abdominal circumference, maternal BMI, and fetal weight percentile (ultrasound). Model 2, named “ft fat”, exclusively incorporated measurements of subcutaneous fat in the fetal arm and thigh. Model 3, termed “ft no fat”, was similar to model 1 but excluded the subcutaneous fat measure. For each of the proposed models, the detection rate (DR) was calculated considering a false positive rate (FPR) = 5, 10, 15, 20%, Area Under the Curve (AUC), and Positive Predictive Value (PPV).

To ensure the interpretability of the classifier models and verify that differences were attributed to the set of features used rather than the classifier itself, Regularized Linear Discriminant Analysis (Shrinkage-LDA) was employed. Model training and validation were conducted using Python 3.8 software with the scikit-learn machine learning library. The data were divided using an 8-way cross-validation strategy, with 70% used for training and 30% for validation. The strategy aimed to maintain a similar number of items per class in both training and validation sets (Figure 2).

## 3. Results

### 3.1. Characteristics of the Study Population

Baseline characteristics were similar between the study groups (Table 1). However, mothers with diabetes showed higher pre-gestational weight and pre-gestational BMI than mothers in the control group (*p* = 0.034 and 0.046, respectively). No significant difference was found between the study groups in biparietal diameter, head circumference, abdominal circumference, femoral length, ratio between male and female fetuses, as well as gestational age at birth. All newborns were evaluated by neonatologists from the institute staff, weight was measured, and somatometry was performed; all had a diagnosis of “normal weight for gestational age,” according to local reference values.

In the group of pregnant diabetic patients, all received counseling from a clinical nutritionist and were assigned a nutritional plan according to their weight, physical activity, and weeks of gestation. Twenty patients (66%) received treatment with metformin (adjusting the dose to achieve the goals of glycemic control), and five (16%) received subcutaneous insulin treatment (adjusting the dose to achieve the goals of glycemic control).

### 3.2. Association between Maternal Diabetes and Fetal Subcutaneous Fat Tissue

**Figure 3:**
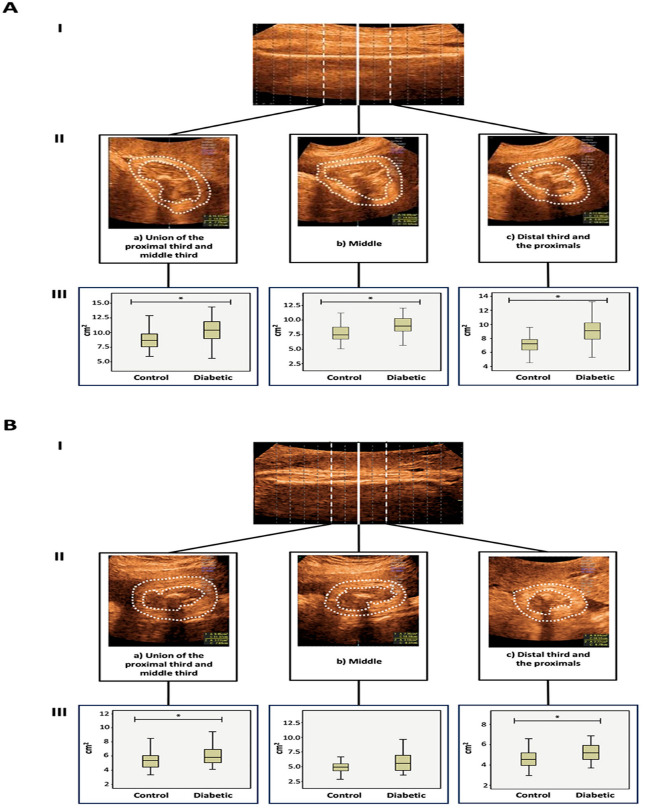
Differences in the fat area around the fetal femur and humerus were analyzed with 3D-View Tomographic Ultrasound Imaging. (**A**) femur; (**I**) Sagittal plane of the femur in the offline analysis. (**II**) Axial plane: Areas corresponding to subcutaneous fatty tissue in offline analysis. (**III**) Boxplots showing comparison between fetuses from diabetic pregnant women vs. healthy pregnant women. (**B**) humerus; (**I**) Sagittal plane of the femur in the offline analysis. (**II**) Axial plane: Areas corresponding to subcutaneous fatty tissue in offline analysis. (**III**) Boxplots showing comparison between fetuses from diabetic pregnant women vs. healthy pregnant women * *p* < 0.05.

The mean fat area (in square centimeters, cm^2^) obtained from six measurements (three from the humerus and three from the femur) was compared between the study groups. A significantly larger fat area was observed in the three selected femur segments of fetuses from women with diabetes than in the control group. These segments included the junction of the proximal third and middle third (*p* = 0.024), the middle third (*p* = 0.026), and the junction of the distal third and middle third (*p* = 0.005) (Table 2 and Figure 3). In the humerus, an increase in fat area was detected at the junction of the proximal third and middle third (*p* = 0.045), as well as at the junction of the distal third and middle third (*p* = 0.023) in fetuses from pregnant women with diabetes, in comparison to healthy controls (Table 2 and Figure 3). When women with pregestational diabetes and gestational diabetes were analyzed separately, no differences were found in the segments evaluated in the fetal arm or thigh (Table 3).

### 3.3. Classifier Models between Fetal Subcutaneous Fat Tissue and Ultrasonographic Tools

Analysis using classifier models to identify whether a patient belonged to the “gestational diabetes” group showed that model 1, which included all the “full model” variables, had a detection rate of 70% considering a false positive rate of 10%, with a positive predictive value of 82%, and an area under the curve of 0.88. Model 2, “ft fat,” had a DR of 38%, considering a false positive rate of 10%, with a PPV of 67% and an AUC of 0.71. Model 3, “ft non-fat,” had a DR of 45%, considering an FPR of 10%, with a PPV of 68% and an AUC of 0.68. The performance of the different models calculated with false positive rates of 5, 10, 15, and 20% are shown in Table 4.

## 4. Discussion

### 4.1. Main Findings

The most striking finding was the significantly larger fat area observed in specific segments of fetuses from mothers with diabetes, regardless of adequate glycemic control compared to the control group. This suggests that maternal diabetes should directly impact the accumulation of subcutaneous fat in certain fetal segments.

### 4.2. Comparison with Existing Literature

This finding is consistent with a prior investigation conducted by Larciprete et al., who utilized ultrasound examinations to illustrate an increase in fetal subcutaneous fat in pregnancies affected by gestational diabetes [27]. However, our study diverges from that research since we exclusively enrolled women with well-controlled diabetes and fetuses of normal weight. In a related study, De Santis et al. in 2010 also documented variations in subcutaneous fat levels among fetuses born to diabetic mothers, highlighting the utility of fat assessment as a third-trimester gestational tool, irrespective of the specific maternal diabetes treatment employed [16]. Building upon their observations, our study concentrated on fetal fat measurements exclusively in the third trimester without stratification by treatment modality.

It is reasonable to assume that the rise in fetal adipose tissue is concomitant with the increase in fetal weight, which is clinically indicative of maternal diabetes decompensation [13]. Therefore, the most noteworthy discovery in our study is the absence of disparities in estimated fetal weight or birth weight between the groups but the increased fat area in the extremities of fetuses born to well-controlled diabetic mothers. Given the insulin sensitivity of adipose tissue, our findings imply that alterations in fetal adipose tissue may function as a more sensitive indicator of the ramifications of maternal metabolic changes, even before significant shifts in fetal weight become apparent [28].

Hence, we can infer that if this cohort of pregnant women with well-controlled diabetes had undergone routine ultrasound assessments without the inclusion of fetal fat measurements, their fetuses would likely have been categorized as having normal weight and presumed to be in good health. This approach, however, would underestimate the metabolic risk associated with changes in body composition. In 2017, Venkataraman et al. provided additional evidence of the “thin but fat” phenotype within the Asian population. They characterized fetuses with a disproportional increase in adipose tissue, even when lean body mass was smaller or comparable, occurring before the biochemical diagnosis of gestational diabetes mellitus. They introduced fetal anterior abdominal wall thickness as an early indicator of this condition [29]. Nevertheless, it is worth noting that this measurement can be influenced by fetal position, orientation, attitude, and the volume of amniotic fluid, potentially reducing its reproducibility. In our study, we assessed the limbs because this approach is not influenced by the variables mentioned earlier. Additionally, the adoption of TUI analysis allows for precise selection of measurement planes, thereby diminishing dependence on inter-observer variability [29,30].

In recent years, there have been notable advancements in ultrasonography, leading to improved resolution. This enhancement enables more precise tissue characterization and accurate quantification of fetal fat accumulation. Additionally, a novel metric called fetal fractional limb volume has emerged, designed to measure the volume of fetal soft tissues, encompassing both fat mass and lean mass [31,32]. It has become evident that substantial physiological diversity and heterogeneity exist in fetal growth velocity patterns, particularly during the third trimester of pregnancy. Furthermore, the growth trajectory of fetal soft tissue volume, primarily comprising fat mass, experiences acceleration in the early stages of the third trimester. Based on these insights, it is suggested that serial assessments of fetal fat mass and fractional limb volume in the third trimester, spaced at intervals of 2–4 weeks, could offer valuable clinical insights. Such assessments have the potential to differentiate between constitutionally small/large fetuses and malnourished/overnourished fetuses, thus facilitating a deeper understanding of the “thrifty” or “drifty” phenotype, both of which are predisposed to the development of metabolic syndrome [33]. By detecting significant variations in fetal fat accumulation, researchers may gain fresh perspectives into the underlying causes of altered fetal body composition observed in conditions such as fetal growth restriction or fetal macrosomia. Further studies must be conducted to evaluate clinical interventions to address altered fetal growth and body composition, with the ultimate goal of primary prevention of future metabolic dysfunction [32,34].

In the forthcoming years, these novel approaches have the potential to reveal that alterations in fetal body composition are equally, if not more, crucial than birth weight alone in identifying newborns with an elevated risk of developing metabolic syndrome, diabetes, heart disease, obesity, and high blood pressure later in life. To rigorously assess this hypothesis, ongoing studies are underway to investigate the influence of changes in fetal body composition on metabolic and neurodevelopmental outcomes in a follow-up cohort at the age of 8 [35,36]. Furthermore, we advocate for the inclusion of comprehensive evaluations at birth and follow-up assessments for fetuses exhibiting growth alterations, such as intrauterine growth restriction and macrosomia. This approach is vital as these fetuses may exhibit similar modifications as previously documented in studies focused on body composition at birth [37,38].

The research also explored the potential of using AI-enhanced classifier models to distinguish between patients with gestational diabetes and those without it. The “full model” achieved a detection rate of 70% at a false positive rate of 10%, indicating a promising ability to identify patients with gestational diabetes.

AI methods in medical care could facilitate individual pregnancy management and improve public health, especially in low- and middle-income countries. Classifier models are one of the methods of analysis that uses AI. Using statistical analysis methods different from those we are conventionally accustomed to seeing in the medical literature is becoming more common to demonstrate the association between variables. Particularly in obstetrics, these analysis methods have been used to evaluate the risk of preeclampsia [39]. We found no history of their use in comorbidities such as diabetes in pregnancy.

### 4.3. Strengths and Limitations

A weakness of our study is the limited number of included patients; however, we assessed the statistical power of the observed differences, all of which exceeded 0.80. Additionally, our study is limited by the exclusion of certain variables that may influence birth weight, such as maternal weight, supplementation, and the use of medications to manage underlying diseases. Nevertheless, existing evidence suggests that various treatments for diabetes do not appear to impact fetal fat measurements.

On the other hand, a strength of our study lies in the comprehensive clinical management provided to all women by the Department of Endocrinology at INPer. Rigorous glycemic control was confirmed through regular measurements of pre- and postprandial capillary and venous blood glucose levels, along with periodic quantification of glycosylated hemoglobin. The employed TUI technique offers the advantage of eliminating operator dependence or bias, as axial cuts are predetermined in the software, accounting for bone edges. The software consistently maintains the same distance between axial planes in all 3D volumes, ensuring consistency across measurements. This contrasts with previous studies that relied on ultrasound-based subcutaneous fat tissue measurement, where operators subjectively selected the measurement plane.

To our knowledge, no other work has utilized classifier models to assess fetal fat measurement as a clinical contributor to diabetes. Thus, this represents the primary strength of our research.

### 4.4. Clinical Interpretation

Fetal weight and the quantity of amniotic fluid are the primary clinical indicators of poorly controlled diabetes during pregnancy. It is worth noting that fetal body composition is also affected in pregnant women with well-controlled diabetes. Consequently, assessing fetal fat content can be a valuable tool, offering advantages over assessing fetal weight alone. This allows clinicians to detect early changes in body composition even before fetal weight is impacted. Detecting such changes during the fetal period provides an opportunity to design and implement early interventions that can positively impact the metabolic control of pregnant women with diabetes, thereby improving perinatal outcomes.

However, establishing reference values for fetal fat is still pending to determine what would be considered normal. Additionally, our findings raise questions about whether the variables currently used in the ultrasonographic evaluation of fetuses from diabetic mothers are adequate or if it is necessary to implement new and more sensitive tools to classify fetuses more accurately at an increased risk of developing metabolic issues later in life [40,41].

The development of a classifier model represents an innovative approach to examining the clinical relevance of subcutaneous fat measurement in fetuses through the utilization of artificial intelligence. In this study, we conducted training and validation of three models. By inputting various variables into the analysis, these models can determine whether the mother–fetus dyad belongs to the diabetic or control group. The analyses of the classifier models indicate that the inclusion of subcutaneous fetal fat measurement via ultrasound leads to a more precise prediction of whether the dyad belongs to the diabetic group. Specifically, this inclusion enhances the detection capability by 10%, raising it from 0.688 to 0.781. These findings support our initial hypothesis that maternal diabetes significantly affects fetal fat.

## 5. Conclusions

This study provides valuable insights into the impact of maternal diabetes on fetal subcutaneous fat tissue. Our findings demonstrate an increase in fat accumulation in fetuses of mothers with well-controlled diabetes. Furthermore, the application of AI-enhanced classifier models allows us to identify the offspring of a diabetic mother. These findings contribute to our comprehension of maternal diabetes and its potential consequences on fetal development, even when the patient is under good glycemic control.

## Figures and Tables

**Table 1:** Clinical characteristics of the population.

	Control *n* = 30	Diabetes *n* = 30	*p* Value
Maternal age (Years, mean ± SD)	30.76 ± 6.4	32.9 ± 7.13	0.247
Gestational age (Weeks, mean ± SD)	34.63 ± 1.7	34.61 ± 1.71	0.609
Pre-gestational maternal weight (kg, ± SD)	69.69 ± 9.7	77.87 ± 15.38	0.034 *
Pre-gestational BMI (kg/m^2^, mean ± SD)	28.33 ± 3.99	31.00 ± 5.20	0.046 *
Parity (Median, minimum, and maximum range)	2 (1–5)	2 (1–6)	0.432
Fetal weight by ultrasound (Grams, mean ± SD)	2447 ± 397	2533 ± 459	0.198
Biparietal diameter (cm)	8.60	8.65	0.322
Cephalic circumference (cm)	30.97	31.26	0.134
Abdominal circumference (cm)	30.57	30.96	0.091
Femoral length (cm)	7.21	6.57	0.074
Newborn weight (Grams, mean ± SD)	3257 ± 298	3389 ± 389	0.233
Gestational age at birth (Weeks, median)	39.1 (37.3–40.1)	38.5 (36.6–39.4)	0.191
Male/female proportion	15/15	15/15	0.478

SD: standard deviation; BMI: body mass index. Student’s *t*-test. * *p* < 0.05.

**Table 2:** Fat area in three axial planes of the femur and humerus among the study groups.

	Control (cm^2^, Mean ± SD) *n* = 30	Diabetes (cm^2^, Mean ± SD) *n* = 30	*p* Value
FEMUR			
Proximal third-middle union	8.9 ± 2.0	10.1 ± 2.0	0.024 *
Middle	7.8 ± 1.7	9.0 ± 2.0	0.026 *
Distal third-middle	7.3 ± 1.7	8.8 ± 1.8	0.005 **
HUMERUS			
Proximal third-middle union	5.4 ± 1.6	6.1 ± 1.4	0.045 *
Middle	5.1 ± 1.4	5.8 ± 1.8	0.069
Distal third-middle	4.7 ± 1.2	5.3 ± 1.2	0.023 *

SD: standard deviation; Paired student’s *t*-test. * *p* < 0.05, ** *p* < 0.01.

**Table 3:** Fat area in three axial planes of the femur and humerus among women with pregestational and gestational diabetes.

	Pregestational Diabetes (cm^2^, Mean ± SD) *n* = 16	Gestational Diabetes (cm^2^, Mean ± SD) *n* = 14	*p* Value
FEMUR			
Proximal third-middle union	9.9 ± 1.6	10.2 ± 1.8	0.34
Middle	8.7 ± 2.1	9.3 ± 2.4	0.42
Distal third-middle	7.9 ± 1.9	8.6 ± 2.1	0.06
HUMERUS			
Proximal third-middle union	5.9 ± 1.8	6.4 ± 1.8	0.35
Middle	5.6 ± 1.2	5.4 ± 1.9	0.69
Distal third-middle	5.1 ± 1.5	5.6 ± 1.4	0.23

SD: standard deviation; Paired student’s *t*-test.

**Table 4:** Performance of the three proposed models: “full model”, “ft-fat”, and “ft-no fat”.

	DR at FPR				AUC	PPV
Model	0.05	0.10	0.15	0.2		
Full model	0.704 (0.214)	0.704 (0.214)	0.738 (0.204)	0.778 (0.182)	0.881 (0.100)	0.823 (0.188)
Ft-fat	0.385 (0.292)	0.385 (0.292)	0.468 (0.279)	0.573 (0.255)	0.719 (0.143)	0.676 (0.190)
Ft no-fat	0.458 (0.269)	0.458 (0.269)	0.501 (0.284)	0.591 (0.269)	0.746 (0.156)	0.682 (0.205)

DR: detection rate; FPR: false positive rate; AUC: area under curve; PPV: positive predictive value. Full model: subcutaneous fat measured by ultrasound, gestational age, fetal weight (ultrasound), fetal abdominal circumference, maternal BMI, and fetal weight percentile (ultrasound); Ft-fat: exclusively incorporated measurements of subcutaneous fat in the fetal arm and thigh; Ft no-fat: excluded the subcutaneous fat measure.

## Data Availability

The data presented in this study are available on request from the corresponding author. The data are not publicly available due to privacy or ethical issues.

## References

[1] Padmanabhan V., Cardoso R.C., Puttabyatappa M. (2016). Developmental Programming, a Pathway to Disease. Endocrinology.

[2] Estrada-Gutiérrez G., Zambrano E., Polo-Oteyza E., Cardona-Pérez A., Vadillo-Ortega F. (2020). Intervention during the first 1000 days in Mexico. Nutr. Rev..

[3] Ornoy A., Reece E.A., Pavlinkova G., Kappen C., Miller R.K. (2015). Effect of maternal diabetes on the embryo, fetus, and children: Congenital anomalies, genetic and epigenetic changes and developmental outcomes. Birth Defects Res. Part C Embryo Today.

[4] Kc K., Shakya S., Zhang H. (2015). Gestational diabetes mellitus and macrosomia: A literature review. Ann. Nutr. Metab..

[5] Jensen L.A., Chik C.L., Ryan E.A. (2016). Review of gestational diabetes mellitus effects on vascular structure and function. Diabetes Vasc. Dis. Res..

[6] Casey B. (2021). Gestational Diabetes—On Broadening the Diagnosis. N. Engl. J. Med..

[7] HAPO Study Cooperative Research Group (2009). Hyperglycemia and Adverse Pregnancy Outcome (HAPO) Study: Associations with neonatal anthropometrics. Diabetes.

[8] Szmuilowicz E.D., Josefson J.L., Metzger B.E. (2019). Gestational Diabetes Mellitus. Endocrinol. Metab. Clin. N. Am..

[9] Alexopoulos A.S., Blair R., Peters A.L. (2019). Management of Preexisting Diabetes in Pregnancy: A Review. JAMA.

[10] Balsells M., García-Patterson A., Gich I., Corcoy R. (2014). Ultrasound-guided compared to conventional treatment in gestational diabetes leads to improved birthweight but more insulin treatment: Systematic review and meta-analysis. Acta Obstet. Gynecol. Scand..

[11] Staud F., Karahoda R. (2018). Trophoblast: The central unit of fetal growth, protection and programming. Int. J. Biochem. Cell Biol..

[12] Godfrey K.M., Costello P.M., Lillycrop K.A. (2016). Development, Epigenetics and Metabolic Programming. Nestle Nutr. Inst. Workshop Ser..

[13] Desoye G., Herrera E. (2021). Adipose tissue development and lipid metabolism in the human fetus: The 2020 perspective focusing on maternal diabetes and obesity. Prog. Lipid Res..

[14] Catalano P.M., Thomas A., Huston-Presley L., Amini S.B. (2003). Increased fetal adiposity: A very sensitive marker of abnormal in utero development. Am. J. Obstet. Gynecol..

[15] Stanirowski P.J., Majewska A., Lipa M., Bomba-Opoń D., Wielgoś M. (2021). Ultrasound evaluation of the fetal fat tissue, heart, liver and umbilical cord measurements in pregnancies complicated by gestational and type 1 diabetes mellitus: Potential application in the fetal birth-weight estimation and prediction of the fetal macrosomia. Diabetol. Metab. Syndr..

[16] de Santis M.S., Taricco E., Radaelli T., Spada E., Rigano S., Ferrazzi E., Milani S., Cetin I. (2010). Growth of fetal lean mass and fetal fat mass in gestational diabetes. Ultrasound Obstet. Gynecol..

[17] Lingwood B.E., Henry A.M., d’Emden M.C., Fullerton A.M., Mortimer R.H., Colditz P.B., KA L.C., Callaway L.K. (2011). Determinants of body fat in infants of women with gestational diabetes mellitus differ with fetal sex. Diabetes Care.

[18] Elessawy M., Harders C., Kleinwechter H., Demandt N., Sheasha G.A., Maass N., Pecks U., Eckmann-Scholz C. (2017). Measurement and evaluation of fetal fat layer in the prediction of fetal macrosomia in pregnancies complicated by gestational diabetes. Arch. Gynecol. Obstet..

[19] Orsso C.E., Silva M.I.B., Gonzalez M.C., Rubin D.A., Heymsfield S.B., Prado C.M., Haqq A.M. (2020). Assessment of body composition in pediatric overweight and obesity: A systematic review of the reliability and validity of common techniques. Obes. Rev..

[20] Sarno L., Neola D., Carbone L., Saccone G., Carlea A., Miceli M., Iorio G.G., Mappa I., Rizzo G., Girolamo R.D. (2023). Use of artificial intelligence in obstetrics: Not quite ready for prime time. Am. J. Obstet Gynecol. MFM.

[21] Borboa-Olivares H., Rodríguez-Sibaja M.J., Espejel-Nuñez A., Flores-Pliego A., Mendoza-Ortega J., Camacho-Arroyo I., González-Camarena R., Echeverría-Arjonilla J.C., Estrada-Gutierrez G. (2023). A Novel Predictive Machine Learning Model Integrating Cytokines in Cervical-Vaginal Mucus Increases the Prediction Rate for Preterm Birth. Int. J. Mol. Sci..

[22] Ramakrishnan R., Rao S., He J.R. (2021). Perinatal health predictors using artificial intelligence: A review. Womens Health.

[23] Markov D. (2008). Tomographic ultrasound imaging (TUI)—Technique and methodological study. Akush. Ginekol..

[24] Rasmussen K.M., Yaktine A.L., National Academies of Sciences Engineering and Medicine (2009). The National Academies Collection: Reports funded by National Institutes of Health. Weight Gain during Pregnancy: Reexamining the Guidelines.

[25] American Diabetes Association (2020). 14. Management of Diabetes in Pregnancy: Standards of Medical Care in Diabetes-2020. Diabetes Care.

[26] Lee W. (2021). Soft tissue assessment for fetal growth restriction. Minerva Obstet. Gynecol..

[27] Larciprete G., Valensise H., Vasapollo B., Novelli G.P., Parretti E., Altomare F., Di Pierro G., Menghini S., Barbati G., Mello G. (2003). Fetal subcutaneous tissue thickness (SCTT) in healthy and gestational diabetic pregnancies. Ultrasound Obstet. Gynecol..

[28] Toro-Ramos T., Paley C., Pi-Sunyer F.X., Gallagher D. (2015). Body composition during fetal development and infancy through the age of 5 years. Eur. J. Clin. Nutr..

[29] Venkataraman H., Ram U., Craik S., Arungunasekaran A., Seshadri S., Saravanan P. (2017). Increased fetal adiposity prior to diagnosis of gestational diabetes in South Asians: More evidence for the ‘thin-fat’ baby. Diabetologia.

[30] Herath M.P., Beckett J.M., Hills A.P., Byrne N.M., Ahuja K.D.K. (2021). Gestational Diabetes Mellitus and Infant Adiposity at Birth: A Systematic Review and Meta-Analysis of Therapeutic Interventions. J. Clin. Med..

[31] Sacks D.A. (1993). Fetal macrosomia and gestational diabetes: What’s the problem?. Obstet. Gynecol..

[32] Ikenoue S., Kasuga Y., Endo T., Tanaka M., Ochiai D. (2021). Newer Insights Into Fetal Growth and Body Composition. Front. Endocrinol..

[33] Sato N., Miyasaka N. (2019). Heterogeneity in fetal growth velocity. Sci. Rep..

[34] Ikenoue S., Akiba Y., Endo T., Kasuga Y., Yakubo K., Ishii R., Tanaka M., Ochiai D. (2021). Defining the Normal Growth Curve of Fetal Fractional Limb Volume in a Japanese Population. J. Clin. Med..

[35] Uthaya S., Bell J., Modi N. (2004). Adipose tissue magnetic resonance imaging in the newborn. Horm. Res..

[36] De Lucia Rolfe E., Modi N., Uthaya S., Hughes I.A., Dunger D.B., Acerini C., Stolk R.P., Ong K.K. (2013). Ultrasound estimates of visceral and subcutaneous-abdominal adipose tissues in infancy. J. Obes..

[37] Lobelo F. (2005). Fetal programming and risk of metabolic syndrome: Prevention efforts for high-risk populations. Pediatrics.

[38] Lee W., Balasubramaniam M., Deter R.L., Hassan S.S., Gotsch F., Kusanovic J.P., Gonçalves L.F., Romero R. (2009). Fractional limb volume--a soft tissue parameter of fetal body composition: Validation, technical considerations and normal ranges during pregnancy. Ultrasound Obstet. Gynecol..

[39] Garcés M.F., Sanchez E., Cardona L.F., Simanca E.L., González I., Leal L.G., Mora J.A., Bedoya A., Alzate J.P., Sánchez Á.Y. (2015). Maternal Serum Meteorin Levels and the Risk of Preeclampsia. PLoS ONE.

[40] Monteiro L.J., Norman J.E., Rice G.E., Illanes S.E. (2016). Fetal programming and gestational diabetes mellitus. Placenta.

[41] Rinaudo P., Wang E. (2012). Fetal programming and metabolic syndrome. Annu. Rev. Physiol..

